# Sociodemographic Factors and Depressive Symptoms Among Cardiac Nurses: A Cross-Sectional Study

**DOI:** 10.3389/fpsyg.2021.723035

**Published:** 2021-08-19

**Authors:** Anna Larysz, Izabella Uchmanowicz

**Affiliations:** ^1^Clinic of Cardiac Transplantation and Mechanical Circulatory Support, Department of Heart Diseases, Wroclaw Medical University, Wroclaw, Poland; ^2^Department of Clinical Nursing, Faculty of Health Sciences, Wroclaw Medical University, Wroclaw, Poland; ^3^Centre for Heart Diseases, Wroclaw Medical University, Wroclaw, Poland

**Keywords:** cardiac nurses, depression, occupational burnout, mood, anxiety, nurses

## Abstract

**Background:** The nursing profession is predisposed toward depressed mood and depressive symptoms. The multidirectionality and intensity of stressors in the nurses' occupational environment are of great significance in this respect. The study aimed to evaluate the impact of selected sociodemographic factors on depressive symptoms among cardiac nurses.

**Methods:** This cross-sectional study included 336 cardiac nurses (302 women and 34 men) and was conducted between December 2019 and September 2020 in four hospital cardiac units in Wroclaw, Poland. Sociodemographic data were collected using a self-developed survey. The following standardized instruments were used for the study outcomes: Patient Health Questionnaire-9 (PHQ-9) and Beck Depression Inventory (BDI).

**Results:** The study among cardiac nurses showed mild depression in 11.61%, moderate depression in 5.06%, and severe depression in 2.68%. Linear regression models showed that significant (*p* < 0.05) predictors of the PHQ-9 score included (1) higher occupational education (bachelor's degree), graduation “only” from medical high school or “other” education; (2) work experience of 16–20 years; (3) living in a relationship; (4) living in a rural area. Linear regression models showed that significant (*p* < 0.05) predictors of the BDI score included (1) higher occupational education (bachelor's degree); (2) graduation “only” from medical high school or “other” education; (3) living in a relationship.

**Conclusions:** Depressive symptoms are a significant problem among Polish cardiac nurses. The prevalence of depressive symptoms is affected by the education level, employment form, marital status, and place of residence.

## Introduction

Depression and mood disorders are serious health problems, especially in developed countries. It is estimated that 12.9% of the worldwide population is affected by depression, with the highest percentage in South America (20.6%) and the lowest in Australia (7.3%) (Lim et al., 2018). In Poland, approximately 3% of the population suffers from depression (Kiejna et al., 2015), and women are more frequently affected than men (Lim et al., 2018).

Due to the incidence of depression in developed countries, the countries have to bear high economic and social costs (Kessler and Bromet, 2013). Economic costs are associated with the treatment of depression, and therefore, approximately PLN 170 million (about EUR 37.8 million) is spent on this purpose in Poland. The social costs are PLN 1–2.6 billion (about EUR 0.22–0.58 billion) annually (Drapała et al., 2014). It was also reported that the annual cost of inpatient care of depression in 2016 was EUR 491 in Poland and EUR 2,848 in Germany (Zaprutko et al., 2018). In addition, there are also indirect costs linked to sickness absenteeism, which is four times more frequent in individuals with depression, and reduced productivity (Cocker et al., 2014).

It is well known that nursing is a profession that especially predisposes to depression (Duan-Porter et al., 2018). The primary reason is the multidirectional character and intensity of occupational stressors (Sarafis et al., 2016; Yoshizawa et al., 2016), coexisted anxiety (Maharaj et al., 2006), and deteriorated psychosomatic wellbeing (Gu et al., 2019), especially considering among frontline nurses in emergency departments during the COVID-19 outbreak (An et al., 2020). Additional predisposing factors include shift work, multi-tasking, constant readiness to deal with a suffering person, a sense of responsibility for the therapeutic process, problems with autonomy in decision-making, a decreasing number of nurses due to staff shortages, and insufficient psychological and financial compensation (Hersch et al., 2016; Kliszcz, 2017). Previous studies have shown that the incidence of depression in early education, already among nursing students, is ~ 24% (Cheung et al., 2016). In contrast, the prevalence of depression among active nurses ranges from 8.8% in Norway (Øyane et al., 2013) to 64.25% in Poland (Kubik et al., 2018).

Anxiety, depression, and aggression, as psychopathological representations of negative affective states, are emotions that are frequently suppressed by highly socialized individuals (Neumann et al., 2010; Compare et al., 2014). The way social roles are fulfilled and factors related to the occupational environment itself may imply the emergence of depressive states. The complexity of the aforementioned biopsychosocial conditions determining the essence of nursing as a profession that overexploits physical and mental potential should be noted. Without a doubt, studies on depression in this professional group are still considered important and necessary. The constantly changing work-related occupational factors and the specificity of nurses' work in a given specialty indicate the need to update scientific evidence and current knowledge. Moreover, there are still limited studies on the subject in relation to nurses who specialize in cardiology. Therefore, this study aimed to evaluate the influence of selected sociodemographic factors on the prevalence of depressive symptoms among cardiac nurses.

## Methods

### Study Design and Settings

This cross-sectional study included 336 cardiac nurses (302 women and 34 men) aged between 20–60 years and was conducted between December 2019 and September 2020 in four hospital cardiac units in Wroclaw, Poland. The Strengthening the Reporting of Observational Studies in Epidemiology (STROBE) guidelines were followed in the design and reporting of this study (von Elm et al., 2007).

### Qualification Procedures

The study group consisted of cardiac nurses working in hospital wards in Wroclaw, Poland. The exclusion criteria included nurses who were not specialized in cardiology, those diagnosed with some prior mental disorder, and those who did not give their informed consent to participate in the study.

### Data Collection

In this study, cardiac nurses from one academic city of Wroclaw were recruited by convenience sampling through personal contact with the research team. The printed questionnaires were prospectively distributed to the group of 469 nurses by their supervisors, who were informed of the eligibility criteria for participation in the study. Supervisors were also responsible for collecting the completed questionnaires. Nurses were informed that participation in the study is voluntary, and they were assured of complete anonymity. In total, 78% of completed questionnaires were returned with 22% missing data. It means that complete data from 366 cardiac nurses were included in the analysis. Researcher Anna Larysz was responsible for verification of the questionnaires' completeness, data collection, and its implementation into the database.

Sample size was calculated by using formula developed for observational and experimental nursing research studies: n = {(Z1–α/2)2 (p) (q)}/d2, where prevalence rate for assessment of depression among nurses was considered 40% (Sharma et al., 2020). Based on data from the Supreme Chamber of Nurses and Midwives, nearly 261,000 nurses were employed in Poland at the time of the study. It was determined that a minimum sample size of 334 completed questionnaires was needed to achieve a confidence level of 95% and that the real value is within ±5% of the confidence interval. The final statistical analysis was performed on data received from 366 study participants.

### Research Tools

Sociodemographic and other related background data were collected using a self-developed survey for sociodemographic data.

#### Patient Health Questionnaire-9 (PHQ-9)

The PHQ-9 is a standardized self-report questionnaire used for screening to diagnose depressive symptoms in the overall population. It ensures both the diagnosis of depression and the evaluation of the severity of its symptoms (Kroenke et al., 2001). The Polish version of the PHQ-9 was translated and validated by Tomaszewski et al. (2011). The PHQ-9 consists of nine main questions and one supplementary question. The answer to each question is scored from 0 to 3, depending on the incidence of a given symptom in the past 2 weeks (3—most frequent symptoms). The maximum score was 27, indicating the highest severity of depressive symptoms. The norm is the score of <5; the range of 5–9 indicates mild depression, 10–14 moderate depression, 15–19 moderately severe depression, and 20–27 severe depression (Tomaszewski et al., 2011).

#### Beck Depression Inventory (BDI)

The BDI is a standardized self-report questionnaire that measures characteristic behaviors and depressive symptoms. The questionnaire consists of 21 questions scored from 0 to 3 points; a higher score indicates more severe symptoms of depression (Beck et al., 1961). Internal consistency for the BDI ranges from 0.73 to 0.92 with a mean of 0.86 (Beck et al., 1988). Thus, the BDI shows high internal consistency, with alpha coefficients of 0.86 and 0.81 for psychiatric and non-psychiatric populations, respectively. This study uses the Polish adaptation by Parnowski and Jernajczyk (1977).

### Ethical Considerations

The study protocol was approved by the local Bioethics Committee of the Wroclaw Medical University, Poland (no. KB−164/2019). All nurses gave their written informed consent before participating in the study. The study was conducted following the Declaration of Helsinki and Good Clinical Practice guidelines.

### Data Analyses

The analysis of quantitative variables (i.e., expressed by number) was conducted by calculating the mean, standard deviation, median, quartiles, minimum and maximum values. The analysis of qualitative variables (i.e., not expressed by number) was conducted by calculating the number and percentage of occurrences of each value. Univariate and multivariate analyses of the effect of multiple variables on the quantitative variable were conducted using linear regression. The results are presented as values of the regression model's parameters with a 95% confidence interval. A significance level of 0.05 was adopted in the analysis. The analysis was conducted using the R program, version 4.0.3 (R Core Team, 2019).

## Results

### Participants' Characteristics

A total of 336 cardiac nurses, including 302 (89.88%) women and 34 (10.12%) men, were included in the study. There were 16.67% nurses from the age group of 20–30, 42.43% nurses from the age group of 31–40, 33.33% nurses from the age group of 41–50, and 28.57% from the group of 51–60. The master's degree in nursing was held by 25% nurses, a bachelor's degree by 30.36% nurses, while as many as 30.95% nurses graduated from medical high school. As many as 54.68% of nurses had the greatest seniority that is more than 20 years. Moreover, the majority of the respondents (52.68%) had one job. Most surveyed nurses, 72.02%, were employed under an employment contract. The most typical monthly amount of working time was 101–180 h, and it concerned 57.74% of nurses. Typical working conditions included teamwork (75.60%) and shift work in the form of 12-h duty shifts (75.0%). The vast majority of respondents were in a relationship (69.94%) and lived in a city > 100,000 residents (63.69%) (Table 1).

**Table 1:** Sociodemographic characteristics of surveyed cardiac nurses.

**Parameter**		**Total (** ***N*** **= 336)**
Age (years)	20–30	56 (16.67%)
	31–40	72 (21.43%)
	41–50	112 (33.33%)
	51–60	96 (28.57%)
Sex	Women	302 (89.88%)
	Men	34 (10.12%)
Education	Medical high school	104 (30.95%)
	Medical vocational school	45 (13.39%)
	Bachelor's degree	102 (30.36%)
	Master's degree	84 (25.00%)
	Other	1 (0.30%)
Job seniority (years)	0–5	54 (16.07%)
	6–10	33 (9.82%)
	11–15	37 (11.01%)
	16–20	28 (8.33%)
	>20	184 (54.76%)
Number of jobs	1	177 (52.68%)
	2	112 (33.33%)
	3	29 (8.63%)
	4	11 (3.27%)
	>5	7 (2.08%)
Employment form	Employment contract	242 (72.02%)
	Contract of mandate/contract for specific work	14 (4.17%)
	Contract	22 (6.55%)
	Employment contract + other form	55 (16.37%)
	Contract + individual practice	2 (0.60%)
	Contract of mandate/contract for specific work + contract	1 (0.30%)
Workload per month (hours)	≤100	5 (1.49%)
	101–180	194 (57.74%)
	181–230	96 (28.57%)
	231–300	31 (9.23%)
	≥301	10 (2.98%)
Nature of work	I work independently	65 (19.35%)
	I work in a group	254 (75.60%)
	I lead a group of employees	17 (5.06%)
System of work	Eight-hour duty	68 (20.24%)
	12-h shift system	252 (75.00%)
	Other	16 (4.76%)
Marital status	Single	66 (19.64%)
	In a relationship	235 (69.94%)
	Widow/widower	13 (3.87%)
Place of residence	City >500,000 residents	214 (63.69%)
	City <100,000 residents	79 (23.51%)
	Rural area	43 (12.80%)

*N, number of participants*.

### Findings of Standardized Tools

The PHQ-9 questionnaire assesses the severity of depression in a respondent. The tool has standards that ensure the interpretation of scores. A total of 141 out of 336 survey participants (41.96%) had no depressive symptoms, 117 respondents (34.82%) suffered from mild depression, 53 (15.77%) had moderate depression, 17 (5.06%) had moderately severe depression, and 8 (2.38%) suffered from severe depression (Table 2).

**Table 2:** Scores and standards for the PHQ-9 and BDI questionnaires.

**Tool**	***N***	**Lack of data**	**Mean**	**SD**	**Median**	**Min**	**Max**	**Q1**	**Q3**
PHQ-9	336	0	6.51	5.19	6	0	27	2	9
BDI	336	0	7.59	7.85	5	0	40	1	12
**Tool**	**Number of points**		**Interpretation**			***n***		**%**	
PHQ-9	0–4		No depression			141		41.96%	
	5–9		Mild depression			117		34.82%	
	10–14		Moderate depression			53		15.77%	
	15–19		Moderately severe depression			17		5.06%	
	20–27		Severe depression			8		2.38%	
BDI	0–13		No depression			271		80.65%	
	14–19		Mild depression			39		11.61%	
	20–28		Moderate depression			17		5.06%	
	29–63		Severe depression			9		2.68%	

*N, number of participants; PHQ-9, Patient Health Questionnaire-9; BDI, Beck Depression Inventory; SD, standard deviation; Min, minimum; Max, maximum; Q1, lower quartile; Q3, upper quartile*.

The BDI provides an assessment of the severity of depressive symptoms in an individual completing the questionnaire. In addition, the BDI has standards that enable the interpretation of the obtained score (Table 2).

No depression was found in 271 out of 336 survey participants (80.65%), mild depression in 39 respondents (11.61%), moderate depression in 17 respondents (5.06%), and severe depression in 9 respondents (2.68%) (Table 2).

### Effects of Sociodemographic Characteristics on the PHQ-9

#### Univariate Analyses

Linear regression models (separate for each of the analyzed characteristics) showed that significant (*p* < 0.05) predictors of the PHQ-9 score include (1) higher vocational education (bachelor's degree): the regression parameter is 1.873 thus it elevates the PHQ-9 score by 1.873 pts on average compared to a master's degree; (2) graduation “only” from medical high school or “other” education: the regression parameter is 1.557 thus it elevates the PHQ-9 score by 1.557 pts on average compared to a master's degree; (3) job seniority of 16–20 years: the regression parameter is −3.21 thus it lowers the PHQ-9 score by 3.21 pts on average compared to job seniority <5 years; (4) living in a relationship: the regression parameter is −2.338 thus it lowers the PHQ-9 score by 2.338 pts on average compared to being single or widowed; (5) living in a rural area: the regression parameter is −1.7, thus it lowers the PHQ-9 score by 1.7 pts on average compared to living in a city >100,000 residents (Table 3).

**Table 3:** Univariate and multivariate analyses for PHQ-9.

**Characteristic**		**Univariate models**				**Multivariate models**			
		**Parameter**	**95% CI**		***p***	**Parameter**	**95% CI**		***p***
Age (years)	20–30	Ref.				Ref.			
	31–40	−1.375	−3.189	0.439	0.138	1.821	−1.598	5.24	0.297
	41–50	−0.714	−2.38	0.952	0.401	3.917	−0.231	8.066	0.065
	51–60	−0.708	−2.42	1.003	0.418	3.365	−0.898	7.627	0.123
Sex	Women	Ref.				Ref.			
	Men	0.703	−1.137	2.544	0.454	0.32	−1.779	2.42	0.765
Education	Master's degree	Ref.				Ref.			
	Bachelor's degree	1.873	0.391	3.355	0.014^*^	2.173	0.621	3.725	0.006^*^
	Medical vocational school	−0.3	−2.158	1.558	0.752	0.844	−1.19	2.878	0.417
	Medical high school, other	1.557	0.085	3.03	0.039^*^	2.301	0.549	4.053	0.011^*^
Job seniority [years]	0–5	Ref.				Ref.			
	6–10	−2.101	−4.334	0.132	0.066	−2.617	−6.151	0.916	0.148
	11–15	−1.483	−3.64	0.673	0.179	−2.613	−6.43	1.204	0.181
	16–20	−3.21	−5.564	−0.857	0.008^*^	−5.96	−10.383	−1.537	0.009^*^
	>20	−1.345	−2.909	0.219	0.093	−4.563	−8.873	−0.253	0.039^*^
Number of jobs	1	Ref.				Ref.			
	2	0.18	−1.046	1.406	0.774	−0.098	−1.663	1.468	0.903
	3	1.993	−0.041	4.027	0.056	1.699	−0.534	3.931	0.137
	>4	0.64	−1.872	3.152	0.618	0.183	−2.437	2.804	0.891
Employment form	Employment contract	Ref.				Ref.			
	Employment contract + other form	0.155	−1.369	1.678	0.842	−1.316	−3.292	0.66	0.193
	Other	−0.09	−1.849	1.67	0.92	−0.862	−2.915	1.191	0.411
Workload per month[hours]	≤180	Ref.				Ref.			
	181–230	0.209	−1.054	1.472	0.746	0.307	−1.251	1.865	0.699
	>230	1.259	−0.484	3.003	0.158	1.404	−0.636	3.445	0.178
Nature of work	I work independently	Ref.				Ref.			
	I work in a group	0.464	−0.952	1.881	0.521	1.014	−0.559	2.587	0.208
	I lead a group of employees	0.199	−2.577	2.975	0.888	1.154	−1.699	4.007	0.429
System of work	8-h duty	Ref.				Ref.			
	12-h shift system	0.261	−1.13	1.651	0.713	−0.239	−1.885	1.407	0.776
	Other	1.765	−1.062	4.592	0.222	1.307	−1.714	4.329	0.397
Marital status	Single	Ref.				Ref.			
	In a relationship	−2.338	−3.64	−1.036	<0.001^*^	−1.833	−3,243	−0.423	0.011^*^
Place of residence	City >100,000 residents	Ref.				Ref.			
	City <100,000 residents	0.532	−0.8	1.863	0.434	0.7	−0.652	2.053	0.311
	Rural area	−1.7	−3.391	−0.01	0.049^*^	−1.387	−3.098	0.324	0.113

* *Statistically significant relationship (p < 0.05)*.

*N, number of participants; CI, confidence interval; p, level of statistical significance; PHQ-9, Patient Health Questionnaire-9; BDI; Ref., referral position*.

#### Multivariate Analysis

The multivariate linear regression model showed that significant (*p* < 0.05) independent predictors of the PHQ-9 score include (1) higher vocational education (bachelor's degree): the regression parameter is 2.173 thus it elevates the PHQ-9 score by 2.173 pts on average compared to master's degree education; (2) graduation “only” from medical high school or “other” education: the regression parameter is 2.301 thus it elevates the PHQ-9 score by 2.301 pts on average compared to a master's degree; (3) job seniority of 16–20 years: the regression parameter is −5.96 thus it lowers the PHQ-9 score by 5.96 pts on average compared to job seniority <5 years; (4) job seniority >20 years: the regression parameter is −4.563 thus it lowers the PHQ-9 score by 4.563 pts on average compared to job seniority <5 years; (5) living in a relationship: the regression parameter is −1.833 thus it lowers the PHQ-9 score by 1.833 pts on average compared to being single or widowed (Table 3).

### Effects of Sociodemographic Characteristics on BDI

#### Univariate Analyses

The linear regression models (separate for each of the analyzed characteristics) showed that significant (*p* < 0.05) predictors of the BDI score include (1) higher vocational education (bachelor's degree): the regression parameter is 3.151; thus, it elevates the BDI score by 3.151 pts on average compared to a master's degree; (2) graduation “only” from medical high school or “other” education: the regression parameter is 4.031 thus so it elevates the BDI score by 4.031 pts on average compared to a master's degree; (3) living in a relationship: the regression parameter is −3.551 thus it lowers the BDI score by 3.551 pts on average compared to being single or widowed (Table 4).

**Table 4:** Univariate and multivariate analyses for BDI.

**Characteristic**		**Univariate models**				**Multivariate models**			
		**Parameter**	**95% CI**		***p***	**Parameter**	**95% CI**		***p***
Age (years)	20–30	Ref.				Ref.			
	31–40	−1.534	−4.276	1.209	0.274	0.217	−4.903	5.337	0.934
	41–50	0.062	−2.456	2.581	0.961	2.562	−3.651	8.774	0.42
	51–60	0.213	−2.375	2.801	0.872	2.362	−4.021	8.746	0.469
Sex	Women	Ref.				Ref.			
	Men	1.01	−1.774	3.794	0.478	2.134	−1.01	5.278	0.184
Education	Master's degree	Ref.				Ref.			
	Bachelor's degree	3.151	0.92	5.381	0.006^*^	3.083	0.758	5.407	0.01^*^
	Medical vocational school	2.542	−0.255	5.339	0.076	3.723	0.677	6.768	0.017^*^
	Medical high school, other	4.031	1.814	6.247	<0.001^*^	4.503	1.879	7.127	0.001^*^
Job seniority [years]	0–5	Ref.				Ref.			
	6–10	0.175	−3.216	3.566	0.919	1.804	−3.488	7.096	0.505
	11–15	−3.253	−6.529	0.022	0.052	−2.945	−8.662	2.771	0.313
	16–20	−0.001	−3.575	3.573	0.999	−1.852	−8.476	4.773	0.584
	>20	−0.189	−2.564	2.186	0.876	−2.989	−9.443	3.465	0.365
Number of jobs	1	Ref.				Ref.			
	2	0.665	−1.198	2.529	0.484	−0.275	−2.62	2.069	0.818
	3	0.884	−2.208	3.975	0.576	0.176	−3.167	3.519	0.918
	>4	0.746	−3.072	4.564	0.702	−0.616	−4.541	3.308	0.758
Employment form	Employment contract	Ref.				Ref.			
	Employment contract + other form	0.962	−1.327	3.251	0.411	−0.056	−3.015	2.904	0.971
	Other	−2.377	−5.022	0.267	0.079	−3.314	−6.388	−0.24	0.035^*^
Workload per month (hours)	≤180	Ref.				Ref.			
	181–230	0.352	−1.563	2.267	0.719	0.539	−1.794	2.871	0.651
	>230	1.023	−1.62	3.667	0.448	1.436	−1.62	4.492	0.358
Nature of work	I work independently	Ref.				Ref.			
	I work in a group	1.891	−0.238	4.02	0.083	1.938	−0.418	4.294	0.108
	I lead a group of employees	−1.039	−5.211	3.134	0.626	−0.003	−4.276	4.269	0.999
System of work	8-h duty	Ref.				Ref.			
	12-h shift system	1.892	−0.206	3.99	0.078	0.682	−1.783	3.147	0.588
	Other	1.787	−2.479	6.053	0.412	0.657	−3.868	5.182	0.776
Marital status	Single	Ref.				Ref.			
	In a relationship	−3.551	−5.519	−1.582	<0.001^*^	−3.42	−5.531	−1.308	0.002^*^
Place of residence	City >100,000 residents	Ref.				Ref.			
	City <100,000 residents	1.015	−0.999	3.029	0.324	1.168	−0.857	1.168	0.259
	Rural area	−2.459	−5.016	0.098	0.06	−2.76	−5.322	−0.198	0.036^*^

* *Statistically significant relationship (p < 0.05)*.

*N, number of participants; CI, confidence interval; p, level of statistical significance; BDI, Beck Depression Inventory; ref., referral position*.

#### Multivariate Analysis

The multivariate linear regression model showed that significant (*p* < 0.05) independent predictors of the BDI score include (1) higher vocational education (bachelor's degree): the regression parameter is 3.083 thus it elevates the BDI score by 3.083 pts on average compared to a master's degree; (2) graduation “only” from medical vocational school: the regression parameter is 3.723 thus it elevates the BDI score by 3.723 pts on average compared to a master's degree; (3) graduation “only” medical high school or “other” education: the regression parameter is 4.503 thus it elevates the BDI score by 4.503 pts on average compared to a master's degree; (4) a form of employment other than an employment contract (solo trader or employment contract combined with another contract): the regression parameter is −3.314 thus it lowers the BDI score by 3.314 pts on average compared to an employment contract; (5) living in a relationship: the regression parameter is −3.42 thus it lowers the BDI score by 3.42 pts on average compared to being single or widowed; (6) living in a rural area: the regression parameter is −2.76 thus it lowers the BDI score by 2.76 pts on average compared to living in a city >100,000 residents (Table 4).

## Discussion

Previous research on the prevalence of depression among active nurses showed wide geographic variation. In highly developed countries, i.e., Norway or Canada, the prevalence is 8.8% and 9.1%, respectively. In the studies conducted in the Philippines, it was shown that depression affects one in five nurses (Batalla et al., 2019), while one in four nurses in Brazil (Schmidt et al., 2011), and one in three active nurses and India (Shajan and Nisha, 2019). The studies conducted in Yoon and Kim (2013), in Japan by Furihata et al. (2020), and in China by Xie et al. (2020) showed that 38.0, 41.7, and 43.83% of nurses, respectively, have depression-related symptoms.

In contrast, a study conducted in Poland by Kubik et al. (2018) showed that depression affects approximately two-thirds of nurses. In this study, the percentage of nurses who were diagnosed with symptoms indicative of depression varied due to a type of screening tool. In the assessment using BDI, <20% of the respondents had symptoms indicating depression, while according to the PHQ-9 scale, almost 60% of the respondents had depression. Due to differences in the tools used for assessing the presence of symptoms indicative of depression, it is complicated to compare the outcomes of the studies. However, some trends can be observed—in highly developed countries where health care funding is significantly higher, the percentage of nurses reporting a depression-related problem is significantly lower. This may be affected by better working conditions and access to professional psychological support, which nurses can use when needed. In the study by Kubik et al. (2018), more than 80% of nurses declared their willingness to use the help of a psychologist or psychotherapist in difficult situations.

The analysis showed that cardiac nurses with education lower than a master's degree were more likely to experience depressive symptoms. This association is consistent with the findings obtained by Ning et al. (2020), who found that depression was more prevalent among younger employees (≤40 years) and medical staff with junior titles. Nurses holding a master's degree may have better job salaries and benefits, so they have less opportunity to worry about Ariapooran (2019).

Ariapooran (2019) and Chiang and Chang (2012) showed that nurses with less seniority reported more depressive symptoms than those with more seniority, which was confirmed in this study in which nurses with more than 20 years of seniority were less likely to experience depressive symptoms than those with less seniority. Nurses with less seniority suffer more severe stress when caring independently for patients with difficult clinical conditions due to less work experience (Ning et al., 2020). It was confirmed that this might translate into an increased risk of depression development (Cheung et al., 2016).

Also, individuals in a relationship had a lower risk of developing depression than those who were single or widowed. This is consistent with the findings of Yoon and Kim (2013) and Ariapooran (2019), according to which single nurses had particularly high levels of depressive symptoms. Our results support the hypothesis that marriage provides greater emotional support to counteract stress reactions among nurses. Beam et al. (2017) showed that marriage reduces the genetic influence of perceived stress on depressive symptoms but does not reduce the influence of environmental factors. Furthermore, marriage may reduce the heritability of depressive symptoms associated with perceived stress. In contrast, Chiang and Chang (2012) and Cheung and Yip (2015) found that married nurses were more likely to have depressive symptoms than those who were single.

This study has also shown that the place of residence of cardiac nurses determined the occurrence of depressive symptoms—individuals living in rural areas had a lower intensity of symptoms indicative of depression. It is possible that communing with nature after work hours and pursuing more active outdoor leisure activities enable nurses to better cope with stressful situations that arise in the workplace.

### Study Limitations

The strengths of this study were the large sample size and the use of standardized instruments to measure symptoms of depression. However, there were several potential limitations in terms of methodological aspects. The main limitation of the study was linked to the data collection methods used, which were considered a one-time point rather than longitudinal. The study enrolled mainly women, which may be its bias. The method for the selection of the nurses was purposive sampling; the study was conducted among cardiac nurses of four hospitals but within one academic center; therefore, the conclusions cannot be generalized to the broader population of cardiac nurses. Also, some variables associated with depression, such as social support, health status, and pre-existing psychiatric disorders, were not examined. Due to the cross-sectional design of the study, causality between depression and other variables was unable to be investigated.

Furthermore, future research should focus on better recognition of depression among cardiac nurses and the entire nursing professional group, which is essential to consider interventions to improve working conditions and reduce important risk factors for depressive symptoms. Moreover, it is crucial to observe the direct impact of depressive symptoms and professional burnout syndrome on the quality of care provided and, above all, on patient safety. Such findings could be useful for preventing mental health problems among nurses.

### Practical Implications

Depression is a common problem among cardiac nurses. Therefore, considering the negative impact of depression on quality of care, health authorities should organize regular screening targeting depression and develop preventive measures to alleviate the risk of depression by providing a timely provision of financial support, online psychological counseling service, or on-site psychological guidance.

## Conclusions

Depression is a widely prevalent condition among Polish cardiac nurses. The prevalence of depressive symptoms is negatively affected by a lower level of education (lower than a master's degree) and living alone, while it is positively affected by more seniority (over 16 years) and living in a rural area.

## Data Availability Statement

The raw data supporting the conclusions of this article will be made available by the authors, without undue reservation.

## Ethics Statement

The studies involving human participants were reviewed and approved by Bioethics Committee of the Wroclaw Medical University, Poland (approval no. KB−164/2019). The patients/participants provided their written informed consent to participate in this study.

## Author Contributions

AL contributed to study conception and design, data acquisition, analysis and interpretation of data, and drafting of the manuscript. IU contributed to study conception and design, analysis and interpretation of data, and critical revision of the manuscript. All authors approved the final version of the manuscript.

## Conflict of Interest

The authors declare that the research was conducted in the absence of any commercial or financial relationships that could be construed as a potential conflict of interest.

## Publisher's Note

All claims expressed in this article are solely those of the authors and do not necessarily represent those of their affiliated organizations, or those of the publisher, the editors and the reviewers. Any product that may be evaluated in this article, or claim that may be made by its manufacturer, is not guaranteed or endorsed by the publisher.

## References

[B1] An Y. Yang Y. Wang A. Li Y. Zhang Q. Cheung T.. (2020). Prevalence of depression and its impact on quality of life among frontline nurses in emergency departments during the COVID-19 outbreak. J. Affect. Disord. 276, 312–315. 10.1016/j.jad.2020.06.047 32871661 PMC7361044

[B2] Ariapooran S. (2019). Sleep problems and depression in Iranian nurses: the predictive role of workaholism. Iran. J. Nurs. Midwifery Res. 24, 30–37. 10.4103/ijnmr.IJNMR_188_17 30622575 PMC6298169

[B3] Batalla V. R. D. Barrameda A. L. N. Basal J. M. S. Bathan A. S. J. Bautista J. E. G. Rebueno M.a.. (2019). Moderating effect of occupational stress on spirituality and depression of registered nurses in tertiary hospital: a structural equation model. J. Adv. Nurs. 75, 772–782. 10.1111/jan.13856 30230002

[B4] Beam C. R. Dinescu D. Emery R. E. Turkheimer E. (2017). A twin study on perceived stress, depressive symptoms, and marriage. J. Health Soc. Behav. 58, 37–53. 10.1177/0022146516688242 28661771 PMC5746173

[B5] Beck A. T. Steer R. A. Carbin M. G. (1988). Psychometric properties of the beck depression inventory: twenty-five years of evaluation. Clin. Psychol. Rev. 8, 77–100. 10.1016/0272-7358(88)90050-5

[B6] Beck A. T. Ward C. H. Mendelson M. Mock J. Erbaugh J. (1961). An inventory for measuring depression. Arch. Gen. Psychiatry 4, 561–571. 10.1001/archpsyc.1961.01710120031004 13688369

[B7] Cheung T. Wong S. Wong K. Law L. Ng K. Tong M.. (2016). Depression, anxiety and symptoms of stress among baccalaureate nursing students in Hong Kong: a cross-sectional study. Int. J. Environ. Res. Public. Health 13, 779–804. 10.3390/ijerph13080779 27527192 PMC4997465

[B8] Cheung T. Yip P. (2015). Depression, anxiety and symptoms of stress among Hong Kong nurses: a cross-sectional study. Int. J. Environ. Res. Public. Health 12, 11072–11100. 10.3390/ijerph120911072 26371020 PMC4586662

[B9] Chiang Y.-M. Chang Y. (2012). Stress, depression, and intention to leave among nurses in different medical units: implications for healthcare management/nursing practice. Health Policy 108, 149–157. 10.1016/j.healthpol.2012.08.027 23017221

[B10] Cocker F. Nicholson J. M. Graves N. Oldenburg B. Palmer A. J. Martin A.. (2014). Depression in working adults: comparing the costs and health outcomes of working when ill. PLoS ONE 9:e105430. 10.1371/journal.pone.0105430 25181469 PMC4152191

[B11] Compare A. Zarbo C. Shonin E. Van Gordon W. Marconi C. (2014). Emotional regulation and depression: a potential mediator between heart and mind. Cardiovasc. Psychiatry Neurol. 2014:324374. 10.1155/2014/324374 25050177 PMC4090567

[B12] Drapała A. Karczewicz E. Zalewska H. Gierczyński J. Gryglewicz J. Sielicki P.. (2014). Depresja—analiza kosztów ekonomicznych i społecznych. Warsaw: Lazarski University.

[B13] Duan-Porter W. Hatch D. Pendergast J. F. Freude G. Rose U. Burr H.. (2018). 12-month trajectories of depressive symptoms among nurses—contribution of personality, job characteristics, coping, and burnout. J. Affect. Disord. 234, 67–73. 10.1016/j.jad.2018.02.090 29522946 PMC6087547

[B14] Furihata R. Saitoh K. Suzuki M. Jike M. Kaneita Y. Ohida T.. (2020). A composite measure of sleep health is associated with symptoms of depression among Japanese female hospital nurses. Compr. Psychiatry 97:152151. 10.1016/j.comppsych.2019.152151 31954287

[B15] Gu B. Tan Q. Zhao S. (2019). The association between occupational stress and psychosomatic wellbeing among Chinese nurses. Medicine 98:e15836. 10.1097/MD.0000000000015836 31145327 PMC6708716

[B16] Hersch R. K. Cook R. F. Deitz D. K. Kaplan S. Hughes D. Friesen M. A.. (2016). Reducing nurses' stress: a randomized controlled trial of a web-based stress management program for nurses. Appl. Nurs. Res. 32, 18–25. 10.1016/j.apnr.2016.04.003 27969025 PMC5159423

[B17] Kessler R. C. Bromet E. J. (2013). The epidemiology of depression across cultures. Annu. Rev. Public Health 34, 119–138. 10.1146/annurev-publhealth-031912-114409 23514317 PMC4100461

[B18] Kiejna A. Piotrowski P. Adamowski T. Moskalewicz J. Wciórka J. Stokwiszewski J.. (2015). The prevalence of common mental disorders in the population of adult Poles by sex and age structure—an EZOP Poland study. Psychiatr. Pol. 49, 15–27. 10.12740/PP/30811 25844407

[B19] Kliszcz D. (2017). Depresja, lek i pielegniarskie zycie. Mag. Pielegniarki Połoznej 4, 4–7.

[B20] Kroenke K. Spitzer R. L. Williams J. B. (2001). The PHQ-9: validity of a brief depression severity measure. J. Gen. Intern. Med. 16, 606–613. 10.1046/j.1525-1497.2001.016009606.x 11556941 PMC1495268

[B21] Kubik B. Jurkiewicz B. Kołpa M. Stepie,ń K. (2018). Nurses' health in the context of depressive symptoms. Med. Stud. 34, 147–152. 10.5114/ms.2018.76876

[B22] Lim G. Y. Tam W. W. Lu Y. Ho C. S. Zhang M. W. Ho R. C. (2018). Prevalence of depression in the community from 30 countries between 1994 and 2014. Sci. Rep. 8:2861. 10.1038/s41598-018-21243-x 29434331 PMC5809481

[B23] Maharaj H. Maharaj D. S. Daya S. (2006). Acetylsalicylic acid and acetaminophen protect against oxidative neurotoxicity. Metab. Brain Dis. 21, 189–199. 10.1007/s11011-006-9012-7 16855872

[B24] Neumann I. D. Veenema A. H. Beiderbeck D. I. (2010). Aggression and anxiety: social context and neurobiological links. Front. Behav. Neurosci. 4:12. 10.3389/fnbeh.2010.00012 20407578 PMC2854527

[B25] Ning X. Yu F. Huang Q. Li X. Luo Y. Huang Q.. (2020). The mental health of neurological doctors and nurses in Hunan Province, China during the initial stages of the COVID-19 outbreak. BMC Psychiatry 20:436. 10.1186/s12888-020-02838-z 32891124 PMC7474327

[B26] Øyane N. M. F. Pallesen S. Moen B. E. Åkerstedt T. Bjorvatn B. (2013). Associations between night work and anxiety, depression, insomnia, sleepiness and fatigue in a sample of Norwegian nurses. PLoS ONE 8:e70228. 10.1371/journal.pone.0070228 23950914 PMC3737208

[B27] Parnowski T. Jernajczyk W. (1977). Inwentarz Depresji Becka w ocenie nastroju osób zdrowych i chorych na choroby afektywne (ocena pilotazowa). Psychiatr. Pol. 11, 417–425. 594229

[B28] R Core Team (2019). R: A Language and Environment for Statistical Computing. R Foundation for Statistical Computing, Vienna, Austria. Available online at: https://www.R-project.org/ (accessed June 1, 2021).

[B29] Sarafis P. Rousaki E. Tsounis A. Malliarou M. Lahana L. Bamidis P.. (2016). The impact of occupational stress on nurses' caring behaviors and their health related quality of life. BMC Nurs. 15:56. 10.1186/s12912-016-0178-y 27708546 PMC5039891

[B30] Schmidt D. R. C. Dantas R. A. S. Marziale M. H. P. (2011). Anxiety and depression among nursing professionals who work in surgical units. Rev. Esc. Enferm. USP 45, 487–493. 10.1590/S0080-62342011000200026 21655802

[B31] Shajan A. Nisha C. (2019). Anxiety and depression among nurses working in a tertiary care hospital in South India. Int. J. Adv. Med. 6, 1611–1615. 10.18203/2349-3933.ijam20194228

[B32] Sharma S. K. Mudgal S. K. Thakur K. Gaur R. (2020). How to calculate sample size for observational and experimental nursing research studies? Natl. J. Physiol. Pharm. Pharmacol. 10, 1–8. 10.5455/njppp.2020.10.0930717102019

[B33] Tomaszewski K. Zarychta M. Bieńkowska A. Chmurowicz E. Nowak W. Skalska A. (2011). Validation of the Patient Health Questionnaire-9 Polish version in the hospitalised elderly population. Psychiatr. Pol. 45, 223–233. 21714211

[B34] von Elm E. Altman D. G. Egger M. Pocock S. J. Gøtzsche P. C. Vandenbroucke J. P.. (2007). Strengthening the reporting of observational studies in epidemiology (STROBE) statement: guidelines for reporting observational studies. BMJ 335, 806–808. 10.1136/bmj.39335.541782.AD 17947786 PMC2034723

[B35] Xie N. Qin Y. Wang T. Zeng Y. Deng X. Guan L. (2020). Prevalence of depressive symptoms among nurses in China: a systematic review and meta-analysis. PLOS ONE 15:e0235448. 10.1371/journal.pone.0235448 32634150 PMC7340293

[B36] Yoon S. L. Kim J.-H. (2013). Job-related stress, emotional labor, and depressive symptoms among korean nurses: job stress and emotional labor with depressive symptoms. J. Nurs. Scholarsh. 45, 169–176. 10.1111/jnu.12018 23470274

[B37] Yoshizawa K. Sugawara N. Yasui-Furukori N. Danjo K. Furukori H. Sato Y.. (2016). Relationship between occupational stress and depression among psychiatric nurses in Japan. Arch. Environ. Occup. Health 71, 10–15. 10.1080/19338244.2014.927345 25148581

[B38] Zaprutko T. Göder R. Kus K. Pałys W. Rybakowski F. Nowakowska E. (2018). The economic burden of inpatient care of depression in Poznan (Poland) and Kiel (Germany) in 2016. PLoS ONE 13:e0198890. 10.1371/journal.pone.0198890 29902259 PMC6001949

